# One anastomosis gastric bypass vs. Roux-en-Y gastric bypass, remedy for insufficient weight loss and weight regain after failed restrictive bariatric surgery

**DOI:** 10.1007/s11695-020-04536-x

**Published:** 2020-04-19

**Authors:** Nathan Poublon, Ibtissam Chidi, Martijn Bethlehem, Ellen Kuipers, Ralph Gadiot, Marloes Emous, Marc van Det, Martin Dunkelgrun, Ulas Biter, Jan Apers

**Affiliations:** 1 Department of Surgery, Franciscus Gasthuis & Vlietland, Locatie Gasthuis, Afdeling Heelkunde, Kleiweg 500, 3045 PM, Rotterdam, The Netherlands; 2 Department of Surgery, Medisch Centrum Leeuwarden, Leeuwarden, The Netherlands; 3 Department of Surgery, Ziekenhuisgroep Twente, Almelo, The Netherlands

**Keywords:** mgb-oagb, Revisional surgery

## Abstract

**Background:**

Failure occurs in up to 60% of the patients that were treated with primary restrictive bariatric operations such as Laparoscopic Adjustable Gastric Banding (LAGB), or restrictive/metabolic operations like Laparoscopic Sleeve Gastrectomy (LSG). Insufficient weight loss and weight regain are the most commonly reported reasons of failure. The aim of this retrospective multicenter study was to compare One Anastomosis Gastric Bypass (OAGB) to Roux-en-Y Gastric Bypass (RYGB) as a revisional procedure in terms of weight loss, procedure time, complication rate and morbidity.

**Methods:**

491 patients operated on between 2012 and 2017 for failed restrictive surgery were included in this study (OAGB (n=185) or RYGB (n=306)). Failure was defined as total weight loss (TWL) less than 25%, excess weight loss (EWL) less than 50% and/or a remaining body mass index (BMI) larger than 40 kg/m2 at two years of follow up. Primary outcome measures were %TWL and % excess BMI loss (EBMIL) at 12, 24 and 36 months of follow-up. Secondary outcomes were procedure time, reduction of comorbidity, early and late complication rate, and mortality.

**Results:**

%TWL was significantly larger in the OAGB group at 12 months (mean 24.1±9.8 vs. 21.9±9.7, p = 0.023) and 24 months (mean 23.9±11.7 vs. 20.5±11.2, p = 0.023) of follow-up. %EBMIL was significantly larger in the OAGB group at 12 months (mean 69.0±44.6 vs. 60.0±30.1, p = 0.014) and 24 months (mean 68.6±51.6 vs. 56.4±35.4, p = 0.025) of follow-up. Intra-abdominal complications (leakage, bleeding, intra-abdominal abscess and perforation) occurred less frequently after revisional OAGB (1.1% vs. 4.9%, p = 0.025). Surgical intervention for biliary reflux (5.4% vs. 0.3%, p < 0.001) was more prevalent in the OAGB group. Surgical intervention for internal herniation (0.0% vs. 4.9%, p = 0.002) was more prevalent in the RYGB group.

**Conclusions:**

This study suggests that OAGB is superior to RYGB as a remedy for insufficient weight loss and weight regain after failed restrictive surgery with more weight loss and a lower early complication rate. To substantiate these findings, further research from prospective randomized controlled trials is needed.

## Introduction

Obesity is a global health problem of increasing proportions and is a major risk factor for premature death and morbidity such as type 2 diabetes mellitus (T2DM), obstructive sleep apnea syndrome (OSAS), hypertension, dyslipidemia and osteoarthritis [1].

Bariatric surgery is the most effective treatment in morbidly obese patients, in terms of weight loss and reduction of these comorbidities [2, 3].

Primary restrictive procedures such as Vertical Banded Gastroplasty (VBG), Laparoscopic Adjustable Gastric Banding (LAGB) or Laparoscopic Sleeve Gastrectomy (LSG), although the latter is considered to be a metabolic procedure, seem to be a safe and effective strategy for weight reduction in short and mid-term follow-up. However, patients who underwent these procedures are at risk of having insufficient weight loss and weight regain [4–8]. These complications can be an indication for revisional surgery [8, 9]. Cumulative failure rates in patients who received LSG and LAGB have been reported up to 30 and 60%, respectively [10–17]. However, revisional procedures appear to be only moderately effective in terms of additional weight loss. On average, revisional surgery has a higher complication rate and longer length of stay compared to primary bariatric interventions [18, 19].

Roux-en-Y gastric bypass (RYGB) is currently the golden standard as a revisional procedure. However, various success rates of RYGB as a remedy of failure of restrictive surgery are reported [20–33]. In current literature weight loss results of revisional RYGB is usually inferior compared to primary RYGB, with an reported additional percentage excess body mass index loss (EBMIL) at 6, 12 and 48 months of 22.5%, 29.1% and 37.5%, respectively [20, 31]. Early complications such as leakage, bleeding and infection occur in 11.8-16.6% of the cases and surgical intervention due to these complications is performed in 4.5-8% [20–23]. A long-term complication of (revisional) RYGB, which often requires surgical intervention, is internal herniation and occurs in 3.3-4.9%. [20, 25, 29].

The benefits of RYGB as a revisional procedure might outweigh the risk for the majority of the patients, but the overall complication rate remains relatively high. Due to technical difficulties of revisional surgery a learning curve is present, but most recent literature still reports an early allcause complication rate of 4.5-11.4% [31–33]. In previous studies on revisional RYGB, series are often small, and groups are heterogeneous, with both weight-related problems and complications of primary surgery-related being an indication for revision.

Therefore, caution is needed to draw firm conclusions about the benefits of revisional RYGB and a more targeted approach in finding a remedy for patients after failed restrictive surgery is paramount.

One Anastomosis Gastric Bypass (OAGB), as first reported by Rutledge [34], has been described as a viable alternative, due to its relative technical simplicity, reversibility and shorter operation time.

Primary OAGB is effective in weight loss and remission of T2DM and superior to RYGB in this respect, while being associated with a shorter operating time and slightly less post-operative complications [35]. As a primary procedure when compared to RYGB, percentage excess weight loss (EWL) five years post-surgery of OAGB has been reported as 72.9% vs. 60.1% with a complication rate of 3.2% vs. 1.8% [36–41]. The prevalence of gastric ulcers between OAGB and RYGB is similar [42–44]. A long-term concern after OAGB is symptomatic biliary reflux gastritis, which requires revisional surgery. More recent trials report a biliary reflux rate of 0.4-0.9% and clear implication of this in clinical practice still needs to be established.

Several studies showed the efficacy of OAGB as a revisional procedure as well, in terms of weight loss and reduction of comorbidity [44–49]. After failed restrictive operations, EBMIL of up to 66% is reported at five years follow up [45]. However, cohorts are small and no comparative research between OAGB and RGYB as a revisional procedure has been conducted up to this point.

The aim of the current study was to compare OAGB to RYGB as a revisional procedure after failed restrictive surgery in terms of weight loss, procedure time, complication rate, reduction of comorbidity, complication rate and mortality.

## Methods

### Study Objective and Design

The primary aim of this retrospective multicenter study was to compare effectiveness of OAGB and RYGB as a remedy for insufficient weight loss and weight regain after failed primary restrictive procedures in terms of additional weight loss. Secondary objectives were to evaluate procedure time, reduction of comorbidity, short- and long term complication rate, and mortality between the two procedures.

### Patient selection and data collection

For this study, selected patients were retrospectively analyzed. Data were collected from hospital medical records. Adult patients who presented with insufficient weight loss and/or weight regain after failed LSG or LAGB after two years and who underwent revisional OAGB or RYGB at one of three Dutch high volume bariatric hospitals from 2012 to 2017, were included in this study. Patients who received revisional surgery for other indications such as comorbidity or complications of primary surgery were excluded from this study. In accordance with IFSO guidelines, all patients were between 18 and 65 years of age when the primary procedure was performed.

Preoperative assessments of patient eligibility for bariatric surgery included consultation with an endocrinologist, a dietician, and a psychologist to exclude patients with non-adjusted eating patterns or eating disorders. If detected, these issues were addressed and fully treated before surgery was considered. Insufficient weight loss or weight regain was defined as EWL <50% and/or TWL <25% and/or BMI >40 kg/m2 at 2 years follow-up. The medical-ethical committees of all participating hospitals approved this study prior to data collection. Exclusion criteria were known psychiatric illness or eating disorder, pregnancy, previous gastric surgery other than primary bariatric procedure.

### Surgical Technique and Perioperative Management

#### One Anastomosis Gastric Bypass

A standardized operation protocol was used in all patients as reported previously [46, 55]. In case the previous procedure consisted of LAGB, the band was removed as well as dissection of the angle of His. The capsule of the band on the stomach was occasionally divided or removed. The linear stapler divides the stomach horizontally at the junction of the corpus and antrum at the level of crow’s foot. In case of a previous gastric sleeve, mostly only horizontal transection was used. Limb length varied from 150 to 250 cm based on the patient’s preoperative BMI. This tailored approach, which was modified from Lee’s recommendations [41, 43], involved a BP limb of 150 cm for patients with BMI 40 and 50.

#### Roux-en-Y Gastric Bypass

A standardized operation protocol was used in all patients. The technique of this procedure is also described by Emous. [55] In case the previous procedure consisted of LGB, the band was removed as well as adhesiolysis of the angle of His. The capsule of the band on the stomach was occasionally divided or removed. After this the bypass was performed. The mesenteric defects were not routinely closed in all centers.

### Postoperative Care

All bariatric patients enrolled in an enhanced recovery program and received 3-12 months of postoperative PPI therapy to prevent marginal ulcers. Patients were advised to quit smoking prior to undergoing OAGB or RYGB. Postoperative daily multivitamin supplements were prescribed. Patients were put on a fluid diet for 2-3 weeks beginning on the first postoperative day. Most patients were discharged from the hospital 1–2 days after surgery. Patients received postoperative counseling by the surgeon or specialized bariatric nurse at 4-8 weeks after surgery, at 12 months after surgery, and yearly thereafter until five years after last surgery. After surgery, all patients were scheduled for a 1.5-year, sequential (monthly) group meeting program that included counseling by a bariatric nurse practitioner, a dietician, a psychologist, and a physiotherapist.

### Outcome Measures

Baseline characteristics before revisional surgery were determined and entered into the database. Each patient’s ideal weight was estimated based on a target BMI of 25. The primary outcome was percentage total weight loss (%TWL) and percentage excess body mass index loss (%EBMIL) at 12, 24, and 36 months of follow-up, measuring the difference between weightrelated parameters before and after performing the revisional procedure. These measurements were performed by doctors or specialized bariatric nurses in one of the participating hospitals. %TWL, %EBMIL, and improvement of comorbidities was defined according to Brethauer et. al. [50]. Secondary outcomes were mortality, complications following revisional surgery (Clavien Dindo grade 3 or higher), operation time and reduction of comorbidity. Complications were divided into short-term (< 30 days) including anastomotic leak, bleeding, infection, perforation, myocardial infarction and stroke and long-term (> 30 days) including nutritional deficiencies, gastric ulceration, stricture and bile reflux, internal herniation and malnutrition. Comorbidities that were investigated included T2DM, CVD, OSAS, dyslipidemia and osteoarthritis.

### Statistical Analysis

Data were analyzed using the Statistical Package for the Social Sciences (SPSS) version 22. Normality of continuous data was tested with the Shapiro-Wilk test. Descriptive statistics were performed to report patient characteristics. For continuous data mean ± SE was calculated or median and percentiles for variables that were not normally distributed. For categorical data frequencies were calculated.

Differences between groups in continuous data will be tested by a two-sided Student’s t test or a Mann-Whitney U test for variables that were not normally distributed. Subgroup analysis will be conducted where applicable. A p-value <0.05 was taken as the threshold of statistical significance.

## Results

### Pre-operative Patient Characteristics

Between January 2012 and September 2017, 495 patients underwent either OAGB (*n* = 189) or RYGB (*n* = 310) as revisional surgery after a failed restrictive procedure. Demographics before revisional surgery are shown in Table 1. Patients receiving OAGB were younger (mean age 46 ± 9.0 vs. 48 ± 9.6, *p* = 0.018) and less often female (75.5% vs. 84.3%, *p* = 0.017). Sleeve gastrectomy was more often performed as primary procedure (35.1% vs. 24.5%, *p* = 0.012) in the OAGB group, and adjustable gastric banding was more often performed (64.9% vs. 75.5%, *p* = 0.012) in the RYGB group. Furthermore, there were no significant differences between groups. In cases where comorbidity was reported, dyslipidemia (19.0% vs. 27.7%, *p* = 0.033) was more frequent in the RYGB group. No differences in frequency of other comorbidities (T2DM, CVD, OSAS, osteoarthritis, and GERD) were found (Table 1).

**Table 1:** Preoperative demographics

	*N*		
	OAGB (*n* = 185)	RYGB (*n* = 306)	*p* value
Female	139 (75.5%)	258 (84.3%)	0.017
Age	46 ± 9.0	48 ± 9.6	0.018
BMI pre-primary surgery	45.7 [41–51]	45.0 [42–49]	0.374
Primary procedure			
Gastric sleeve	65 (35.1%)	75 (24.5%)	0.012
Adjustable gastric banding	120 (64.9%)	231 (75.5%)	0.012
Indication for revisional surgery			
Insufficient weight loss	57 (30.8%)	89 (29.1%)	0.685
Weight regain	105 (56.8%)	163 (53.3%)	0.452
Insufficient weight loss and weight regain	23 (12.4%)	54 (17.6%)	0.124
BMI pre-revisional surgery	40.9 [36–45]	40.2 [37–45]	0.730
Comorbidity			
T2DM	36/175 (20.6%)	63/306 (20.6%)	0.997
CVD	52/172 (30.2%)	96/305 (31.5%)	0.778
OSAS	20/177 (11.3%)	23/306 (7.5%)	0.160
Dyslipidemia	33/174 (19.0%)	82/296 (27.7%)	0.033
Osteoarthritis	19/87 (21.8%)	52/249 (20.9%)	0.851
GERD	9/88 (10.2%)	32/252 (12.7%)	0.540

*BMI* body mass index, *T2DM* type 2 diabetes mellitus, *CVD* cardiovascular disease, *OSAS* obstructive sleep apnea syndrome, *GERD* gastro-esophageal reflux disease

Data are displayed as mean ± SE, median and percentiles or frequencies and percentage

### Perioperative Details and Early Complications

Procedure time was significantly shorter in the OAGB group (median time 72 [56–95] minutes vs. 83 [66–103] minutes, *p* < 0.001). Compared with RYGB, length of biliary limb was 80 cm longer on average in the OAGB group (median length 180 [175–180] vs. 100 [80–150], *p* < 0.001). Early complication rate (< 30 days post-redo) is listed in Table 2. Significantly less intra-abdominal complications (leakage, bleeding, intra-abdominal abscess, and perforation) were present after revisional OAGB compared with RYGB (1.1% vs. 4.9%, *p* = 0.025). No significant difference in other early complications was present between groups. Two patients died in the follow-up period, one in the OAGB group (0.5%) and one in the RYGB group (0.3%), and this difference was not statistically significant. One patient committed suicide and one patient died due to abdominal sepsis after an incarcerated umbilical hernia, both not attributable to bariatric surgery.

**Table 2:** Intraoperative variables and early complications

	*N*		
	OAGB (*n* = 185)	RYGB (*n* = 306)	*p* value
Length of biliary limb (cm)	180 [175–180]	100 [80–150]	< 0.001
Operation time (min)	72 [56–95]	83 [66–103]	< 0.001
Early complications			
All cause intra-abdominal	2 (1.1%)	15 (4.9%)	0.025
Leakage	1 (0.5%)	2 (0.7%)	0.876
Bleeding	1 (0.5%)	8 (2.6%)	0.295
Intra-abdominal abscess	0 (0.0%)	1 (0.3%)	0.436
Perforation	0 (0.0%)	4 (1.3%)	0.118
Wound infection	5 (2.7%)	5 (1.6%)	0.513
Pneumonia	0 (0.0%)	1 (0.3%)	0.436
Sepsis	0 (0.0%)	1 (0.2%)	0.109
Myocardial infarction	0 (0.0%)	0 (0.0%)	NA
CVA	0 (0.0%)	0 (0.0%)	NA
Re-admittance	4 (2.2%)	6 (2.0%)	0.422
Mortality	1 (0.5%)	1 (0.3%)	0.719

*CVA* cerebrovascular accident

Data are displayed as mean ± SE, median and percentiles or frequencies and percentage

### Weight Loss

Annual BMI and weight loss parameters after revisional surgery are shown in Table 3. No significant difference in BMI was found 12 months post-redo, but BMI was significantly lower in the OAGB group at 24 months (mean 30.8 ± 5.2 vs. 32.6 ± 5.9, *p* = 0.016) and 36 months (mean 31.1 ± 5.1 vs. 34.3 ± 7.1, *p* = 0.012). %TWL was significantly larger in the OAGB group at 12 months (mean 24.1 ± 9.8 vs. 21.9 ± 9.7, *p* = 0.023) and 24 months (mean 23.9 ± 11.7 vs. 20.5 ± 11.2, *p* = 0.023) of follow-up.

**Table 3:** Follow-up weight (total)

Months after redo	Mean ± SD/median (range)		
	OAGB	RYGB	*p* value
12 months	*n* = 154	*n* = 267	
BMI	30.7 [27–34]	31.6 [28–35]	0.057
TWL	24.1 ± 9.8	21.9 ± 9.7	0.023
EBMIL	69.0 ± 44.6	60.0 ± 30.1	0.014
24 months	*n* = 90	*n* = 174	
BMI	30.8 ± 5.2	32.6 ± 5.9	0.016
TWL	23.9 ± 11.7	20.5 ± 11.2	0.023
EBMIL	68.6 ± 51.6	56.4 ± 35.4	0.025
36 months	*n* = 40	*n* = 87	
BMI	31.1 ± 5.1	34.5 ± 7.1	0.009
TWL	22.5 ± 15.2	17.4 ± 13.3	0.056
EBMIL	58.3 ± 36.0	46.5 ± 35.0	0.084

*BMI* body mass index, *TWL* total weight loss %, *EBMIL* excess body mass index loss %

Data are displayed as mean ± SE or median and percentiles

%EBMIL was significantly larger in the OAGB group at 12 months (mean 69.0 ± 44.6 vs. 60.0 ± 30.1, *p* = 0.014) and 24 months (mean 68.6 ± 51.6 vs. 56.4 ± 35.4, *p* = 0.025) of follow-up. %TWL (22.5 ± 15.2 vs.17.4 ± 13.3, *p* = 0.056) and EBMIL% (mean 58.3 ± 36.0 vs. 46.5 ± 35.0, *p* = 0.084) were higher in the OAGB group at 36 months of follow-up as well, but these differences were not statistically significant.

### Late Complications, Morbidity, and Surgical Intervention

Late complications (> 30 days post-redo) are listed in Table 4. No significant difference in the cumulative rate of surgical intervention for any cause due to long-term (> 30 days) complications was found between groups (OAGB vs. RYGB: 9.2% vs. 12.4%, *p* = 0.227). Two patients needed treatment in the intensive care, both in the RYGB group. Surgical intervention due to biliary reflux (5.4% vs. 0.3%, *p* < 0.001) was more prevalent in the OAGB group. Surgical intervention due to internal herniation (0.0% vs. 4.9%, *p* = 0.002) was more prevalent in the RYGB group. No significant difference between the rate of cholecystectomy, iron deficiency, hypoglycemia, hypoalbuminemia, gastric ulcer, or stricture formation of the anastomosis was found between groups. No significant differences in reduction of comorbidities (T2DM, CVD, OSAS, dyslipidemia, osteoarthritis, and GERD) were found (Table 5).

**Table 4:** Follow-up and late complications

	*N*		
	OAGB (*n* = 185)	RYGB (*n* = 306)	*p* value
Surgical intervention (all cause)	17 (9.2%)	38 (12.4%)	0.272
Biliary reflux	22 (11.9%)	6 (2.0%)	< 0.001
For which intervention was needed	10 (5.4%)	1 (0.3%)	< 0.001
Internal herniation	1 (0.5%)	17 (5.6%)	0.004
For which intervention was needed	0 (0.0%)	15 (4.9%)	0.002
Malnutrition	9 (4.9%)	2 (0.7%)	0.002
For which intervention was needed	2 (1.1%)	1 (0.3%)	0.299
Abdominal pain	8 (4.3%)	26 (8.5%)	0.078
For which intervention was needed	3 (1.6%)	14 (4.6%)	0.083
Laparoscopic cholecystectomy	8 (4.3%)	15 (4.9%)	0.769
Iron deficiency	35 (18.9%)	56 (18.3%)	0.236
Hypoglycemia	6 (3.2%)	10 (3.3%)	0.988
Hypoalbuminemia	3 (1.6%)	6 (2.0%)	0.786
Ulcer	4 (2.2%)	6 (2.0%)	0.878
Stricture formation of anastomosis	1 (0.5%)	2 (0.7%)	0.432

Data are displayed as frequencies and percentage

**Table 5:** Post-operative reduction of comorbidities

Comorbidity	*N*		
	OAGB	RYGB	*p* value
T2DM	31/32 (96.9%)	52/62 (83.9%)	0.063
CVD	36/40 (90.0%)	69/87 (79.3%)	0.139
OSAS	14/16 (87.5%)	14/19 (73.7%)	0.309
Dyslipidemia	12/15 (80.0%)	40/45 (88.9%)	0.380
Osteoarthritis	14/19 (73.7%)	44/50 (88.0%)	0.147
GERD	7/9 (77.8%)	27/32 (84.4%)	0.642

*BMI* body mass index, *T2DM* type 2 diabetes mellitus, *CVD* cardiovascular disease, *OSAS* obstructive sleep apnea syndrome, *GERD* gastro-esophageal reflux disease

Data are displayed as frequencies and percentage

## Discussion

In this large multi-center study, OAGB seems more successful in terms of weight loss in the short and mid-term (two years of follow-up) when compared to RYGB. OAGB has a shorter procedure time and a lower complication rate up to thirty days after surgery. No difference is seen in surgical re-interventions and reduction of comorbidity after up to three years of follow up between OAGB and RYGB.

To our knowledge, this is the first large study that compares and describes OAGB versus RYGB as revisional procedure in a large multicenter cohort.

Several studies already showed the effectiveness of OAGB as a primary procedure with noninferior weight loss and a lower complication rate when compared to primary RYGB [24–39]. This study further substantiated the findings of previous publications, through a comparative design and a high-volume multicenter cohort investigating the effectiveness and safety of RYGB and OAGB as a revisional procedure. Thus providing limited evidence that OAGB is more effective for additional weight-loss and could be a safer alternative to RYGB after failed primary restrictive bariatric surgery.

Symptomatic bile reflux, requiring revisional surgery, has been reported as a complication of OAGB [42–44]. Surgical intervention due to bile reflux after redo-OAGB was found in this study as well. The bile reflux rates are relative high compared to those reported in literature, suggesting a possible learning curve is still involved. However, all bariatric surgeons in this study were experienced with OAGB [46] and have passed their learning curve. Another probable reason for this higher rate was a low threshold to convert one anastomosis gastric bypass to RYGB in case of suspicion of bile reflux. It could also be possible that bile reflux incidence is underreported in other studies. The diagnostic workup of bile reflux was not standardized in this trial and could be based on history taking alone. In cases where endoscopy was performed, no difference was found in this study in gastric ulcer formation after OAGB compared to RYGB, as has been described by previous studies before [42–44]. The more recent YOMEGA trial reported more reflux in the gastric pouch in revisional OAGB vs RYGB, without difference in quality of life. [35] A more recent trial failed to detect this difference altogether. [51]

Another concern, which was also found in the YOMEGA trial, are nutritional adverse events after OAGB [35]. In our study we found more malnutrition in the OAGB group as well, but no difference in hypoalbuminemia and no deficiencies that lead to re-intervention. The YOMEGA trial did not report on this and had a non-inferiority design.

Also, BP limb length was on average 200cm in the YOMEGA trial and 180cm in our study which also could explain difference found in the frequency of nutritional adverse events. Theoretically a longer BP limb length, as is common in OAGB when compared to RYGB, could explain the larger weight loss found in OAGB as well as the possibility of more nutritional adverse events. Another recent study, which also had a mean BP length of 200cm, did not find significant differences in nutritional status after revisional OAGB vs revisional RYGB, but did find a better resolution of metabolic syndrome after OAGB. [51] Hence this subject still remains a matter of debate.

High surgical revision rate after revisional RYGB (for internal herniation and abdominal pain) was observed in our study. Preventive closure of mesenteric defects with staples was not routinely performed but gradually became more common practice in the period this trial was conducted and this might have influenced results. The intervention rates due to internal herniation might have been lower if mesenteric defects were closed during all the revisional procedures, as is currently common practice in the participating centers. From earlier research it is known that the percentage of internal herniation could be reduced by halve in case of closure during gastric bypass surgery. Theoretically, if the same would have happened in our study the surgical intervention rate after both procedures could have been comparable. However more recent studies report an incidence of internal herniation of 3.4-4.2% after revisional laparoscopic RYGB, for which intervention was needed, which is still higher than the incidence we found after revisional OAGB. [52, 53]

Thus, we conclude that OAGB as a revisional procedure is at least as safe as RYGB concerning the development of late complications for which intervention is needed. No differences in reduction in comorbidity after up to three years of follow up between OAGB and RYGB. These findings are in line with results from other high-volume comparative trials. [54, 55]

One of the limitations of this study is relative heterogeneity of cohorts due to the retrospective nature of the data collection. Patients were younger and more often male in the OAGB group. Therefore, both selection and reporting bias might have been present and randomized controlled trials are indicated to extrapolate the results of this study to the general population. Another heterogeneity is the difference in primary restrictive procedure that was performed. Sleeve gastrectomy was more prevalent in the OAGB group, gastric banding was more prevalent in the RYGB group. However, this difference didn’t reflect in a difference in BMI, percentage of weight regain or percentage of insufficient weight loss between groups. Thus groups were comparable regarding weight-related parameters at baseline.

Therefore, we conclude that this heterogeneity in the performed primary procedure did not influence the positive results regarding the efficacy of OAGB in terms of weight loss. Our analysis shows superior weight loss in the OAGB group at 12 and 24 months of follow up compared to the RYGB group. Technical differences between revisional surgery after sleeve gastrectomy vs. gastric banding could have biased the lower early complication rate we found after OAGB compared to RYGB.

Another limitation is loss to follow up in medical records of long-term weight loss parameters and comorbidities. This is a well-known phenomenon after bariatric surgery and the reason of this is not completely understood. [56]

In conclusion, the assumption is made that the abovementioned differences between groups did not significantly influence the primary outcome of this trial that OAGB is more effective as a remedy for insufficient weight loss and weight regain after restrictive surgery.

However, more evidence from prospective studies with standardized diagnostic workup of comorbidities and perioperative protocols and randomized groups is needed to substantiate our findings.
